# Recognising and reacting to angry and happy facial expressions: a diffusion model analysis

**DOI:** 10.1007/s00426-018-1092-6

**Published:** 2018-09-07

**Authors:** Jason Tipples

**Affiliations:** Psychology Group, School of Social, Psychological & Communication Sciences, Leeds Beckett University, [CL 815], City Campus, Leeds, LS1 3HE UK

## Abstract

Researchers have reported two biases in how people recognise and respond to angry and happy facial expressions: (1) a gender-expression bias (Becker et al. in J Pers Soc Psychol, 92(2):179–190, 10.1037/0022-3514.92.2.179, 2007)—faster identification of male faces as angry and female faces as happy and (2) an approach–avoidance bias—faster avoidance of people who appear angry and faster approach responses people who appear happy (Heuer et al. in Behav Res The, 45(12):2990–3001, 10.1016/j.brat.2007.08.010 2007; Marsh et al. in Emotion, 5(1), 119–124, 10.1037/1528-3542.5.1.119, 2005; Rotteveel and Phaf in Emotion 4(2):156–172, 10.1037/1528-3542.4.2.156, 2004). The aim of the current research is to gain insight into the nature of such biases by applying the drift diffusion model to the results of an approach–avoidance task. Sixty-five participants (33 female) identified faces as either happy or angry by pushing and pulling a joystick. In agreement with the original study of this effect (Solarz 1960) there were clear participant gender differences—both the approach avoidance and gender-expression biases were larger in magnitude for female compared to male participants. The diffusion model results extend recent research (Krypotos et al. in Cogn Emot 29(8):1424–1444, 10.1080/02699931.2014.985635, 2015) by indicating that the gender-expression and approach–avoidance biases are mediated by separate cognitive processes.

**Electronic supplementary material:**

The online version of this article (10.1007/s00426-018-1092-6) contains supplementary material, which is available to authorized users.

## Introduction

Emotional expressions convey important information about another person—how they are feeling and what they intend to do. Two facial expression processing biases are the focus of the current research—the gender-expression recognition bias (Becker, 2017; Heuer, Rinck, & Becker,^2007^) and the approach–avoidance bias for facial expressions (Heuer et al., 2007; Marsh, Ambady, & Kleck, 2005; Rotteveel & Phaf, 2004). The novelty of the present study is that I apply the diffusion model (Ratcliff, 1978; Wagenmakers, van der Maas, & Grasman, 2007) to understand how these effects might differ.

### Approach–avoidance to facial expressions

Our reactions to seeing someone express anger or happiness are thought to arise rapidly, tapping into our fundamental human motivation to avoid things we dislike and approach things we like (Carver & White, 1994; Davidson, 1998; Gray & McNaughton, 2000; van Peer et al., 2007). Researchers have designed several tasks to measure such processes. In one task (Marsh et al., 2005; Seidel, Habel, Kirschner, Gur, & Derntl, 2010) participants are required to push a joystick away from (avoidance) and pull it toward (approach) themselves in response to specific facial expressions. Instructions to either push or pull in response to a specific expression (e.g., pull for happy and push for angry) are typically varied between blocks. For example, Siedel and colleagues (Seidel et al., 2010) paired expressions within blocks so that participants responded to happy and disgusted facial expression in one block and angry and sad expressions in another block. Although the effects were generally small in magnitude, Siedel et al. recorded an approach effect for happy faces—participants were faster to pull vs push the joystick—and, an avoidance effect for angry faces—participants were faster to push than pull the joystick in response to angry faces.

In keeping with the variable nature of such effects, other studies have also reported considerable variation in the size of the approach–avoidance effect. Key variables that affect both the direction and magnitude of the effect include: individual differences (e.g., Heuer et al., 2007; Struijs et al., 2017), the choice of comparison expression (Paulus & Wentura, 2016), the use of explicit (vs implicit) instructions (Phaf, Mohr, Rotteveel, & Wicherts, 2014), the imagined aggressive intent of the movement in response to the expression (Krieglmeyer & Deutsch, 2013), the evaluative meaning of the response labels (Eder & Rothermund, 2008), social group membership (Paulus & Wentura, 2014), choice of task (Krieglmeyer & Deutsch, 2010) and the type of data transformation and the selection of specific RT cut-offs (Krieglmeyer & Deutsch, 2010). Participant gender differences are less consistently recorded (Krieglmeyer & Deutsch, 2013; Marsh et al., 2005; Seidel et al., 2010; Veenstra, Schneider, Bushman, & Koole, 2017) although the pioneering study of approach–avoidance reactions (Solarz, 1960) reported a larger approach–avoidance effect for female participants in response to emotion words. Overall, these results highlight both the malleability of the effects and a key role for individual differences in approach–avoidance reactions to facial expressions and other affective stimuli.

### Gender-expression recognition bias

The gender-expression bias refers to the finding that people are typically faster and more accurate when categorising male (vs female) faces as “angry” and female (vs male) faces as “happy”. This effect has been reported concurrently with the approach–avoidance effect (Rotteveel & Phaf, 2004) as well as in a separate line of research (Becker, 2017; Becker, Kenrick, Neuberg, Blackwell, & Smith, 2007). Several ideas have been put forward to explain the effect of facial gender on facial expression recognition (for a review see; Adams, Hess, & Kleck, 2015). According to one account, throughout our evolutionary history males and females differed in terms of threats and opportunities and this has helped shape the gender-expression bias. Specifically, throughout evolutionary history males had the potential to hurt—they were threatening—whereas women had the potential for more positive, caring encounters. These regularities are thought to have shaped our perceptual systems to the extent that facial features of typical maleness (e.g., a low brow greater facial angularity) are sufficient to be perceived as threatening. Put differently, recognising facial expression is built on the perceptual processes required for an evolutionary older system—recognising facial gender.

### Rationale

Do the gender-expression and approach–avoidance biases for facial expressions stem from different processes? These biases are rarely studied together but we might expect them to engage separate processes. The approach–avoidance bias is an action bias that we might reasonably expect to be linked to our motor systems whereas, current understanding of the gender-expression recognition bias is that it is based on basic perceptual process (Becker, 2017). Testing such ideas based solely on mean correct reaction times and accuracy rates is challenging because multiple processes might give rise to faster reaction times and increased accuracy. One solution (Krypotos, Beckers, Kindt, & Wagenmakers, 2015) is to apply a cognitive process model for example, the drift diffusion model (for reviews see: Ratcliff & McKoon, 2008; Wagenmakers et al., 2007).

The diffusion model accounts for the distribution of RTs and choice responses in terms of four key psychological processes: (1) evidence accumulation, (2) response bias, (3) response caution and (4) non-decision time. The central idea is that information or evidence is continuously sampled until it reaches a threshold or decision boundary and a response is initiated. The decision boundaries represent the two response options in a binary decision task for example, the “pull” and “push” responses in studies of approach–avoidance. The rate of evidence accumulation toward the boundary is modelled as the drift rate. If the quality of evidence is good then evidence will accumulate rapidly, and this will be indexed by a higher drift rate. For some decisions people will be biased toward making a specific decision before evidence accumulation. This a priori bias in favour of one decision is modelled as a shift in the starting point (*z*) parameter of the diffusion processes. Finally, non-decision time refers to the time taken to encode the stimulus and execute a response—time before and after the diffusion process.

A recent study (Krypotos et al., 2015) used a hierarchical Bayesian version of the diffusion model (Vandekerckhove, Tuerlinckx, & Lee, 2011; Wiecki, Sofer, & Frank, 2013) to model to data from two approach–avoidance studies that used conditioning procedures. An advantage of the hierarchical approach is that it makes maximal use of all the information within a single model—this is particularly advantageous when the number of observations per cell of the design are low. In both studies approach–avoidance reactions were recording using a manikin task in which participants moved a virtual manikin towards and away from either threat (Experiment 1) or appetitively (Experiment 2) conditioned stimuli. Krypotos et al. (2015) reported faster evidence accumulation—higher drift rates—when participants approached appetitive and avoided aversive stimuli. In summary, their findings indicate that for conditioned stimuli, differences in evidence accumulation mediate the effects of threatening and appetitive stimuli on approach–avoidance reactions.

### Summary

Here I used the diffusion model to model data from a study in which participants were required to push and pull a joystick in response to happy and angry facial expressions. The diffusion model can provide a richer account of data from this task. Faster RTs can reflect either increased evidence accumulation or reduced decision boundary separation (lowered caution) or faster response execution. Therefore, the model can provide insight into the processes responsible for the approach–avoidance and gender-recognition biases.

### Predictions RT analyses

Following previous research that used happy and angry expressions I expect to record both the gender-expression and approach–avoidance biases. Specifically, key predictions are: (1) an approach–avoidance bias with faster pull (vs push) responses to happy expressions and conversely, faster push (vs pull) responses to angry expressions and (2) a gender-expression bias namely faster response to male (vs female) angry expressions and conversely faster responses to female (vs male) happy expressions.

### Participant gender

Following Solarz (1960), I included participant gender as a possible correlate of the approach–avoidance and gender-expression RT biases. Also, participants completed a self-reported measure of trait approach–avoidance tendencies namely, the Behavioral Inhibition/Behavioral Activation (BIS/BAS) Scales (Carver and White, 1994). The Behavioral Inhibition and Behavioral Activation systems are thought to underlie patterns of behaviour associated with increased emotion and emotional disorders. For example, increased avoidance in anxiety disorders may be mediated by activity in the neural systems underpinning the Behavioural Inhibition System (for a review see; Bijttebier, Beck, Claes, & Vandereycken, 2009). Although studies have typically failed to record a correlation between trait approach–avoidance and reaction time measures (van Peer, Roelofs, Rotteveel, van Dijk, Spinhoven, & Ridderinkhof, 2010; Struijs et al., 2017) I have included this measure to enable comparison of my sample with others in this area of research field. Moreover, although previous studies have often failed to replicate Solarz’s participant gender effect, they typically have not concurrently measured trait approach–avoidance (for an exception see Struijs et al., 2017). Females typically score higher than males on the measures of Behavioural Inhibition such as the BIS scale (Jorm et al., 1998) and therefore, if an effect is weak or absent it is important to establish whether the sample includes individuals with similar BIS and BAS scores to those in other studies. In short, I tested for participant gender differences and took measures of Behavioural Inhibition and Behavioural Approach.

### Predictions diffusion model

For the diffusion model, the first prediction from previous research (Krypotos et al., 2015) is that evidence accumulation will also mediate approach–avoidance responses to angry and happy faces. Specifically, the prediction is that drift rates will be higher for angry expression when participants are instructed to push compared to pull—the avoidance response—and conversely, drift rates will be higher when participants are required to pull compared to push for happy expressions—the approach response. The approach–avoidance effect is typically conceived as an action bias and therefore, one possibility is that the diffusion model parameter thought to reflect response execution—non-decision times—might be reduced when participants are required to make either an approach or avoidance-compatible response. For the gender-expression bias, recent research from an evolutionary perspective (Becker, 2017) supports a perceptual basis for the influence of gender on angry and happy gender decisions. In the diffusion model, perceptual processes are modelled as influencing the drift rate and non-decision times. Therefore, drift rates might be higher and non-decision times faster for both angry-male and happy-female faces compared to their opposite sex pairs.

### Modelling procedure

Recent research (van Ravenzwaaij, Donkin & Vandekerckhove, 2017) indicates that a computationally simplified version of the diffusion model—the EZ-diffusion model (Wagenmakers et al., 2007)—can provide good estimate of the key diffusion model parameters. The EZ approach reduces the full Ratcliff DDM to 3 parameters (drift rate, boundary separation and non-decision time) by omitting trial-by-trial variability in the model parameters and by assuming a symmetric starting point, *z* = *a*/2. The EZ model parameters are calculated for each person in each condition from the RT mean, RT variance, and percentage correct. The EZ model has provided insight into the psychological process underlying several effects including the effects of alcohol on perceptual decision-making (van Ravenzwaaij, Dutilh & Wagenmakers, 2012) and how aging correlates with perceptual decision-making (Schmiedek, Lövdén, & Lindenberger, 2009). Moreover, the EZ-diffusion model parameters show equivalent retest reliability to the more computationally intensive maximum likelihood fitting procedure (Lerche & Voss, 2017).

## Method

### Compliance with ethical standards

This research was carried out in accordance with the ethical standards of the British Psychological Association and with the 1964 Helsinki declaration and its later amendments. Informed consent was obtained from all participants in the study. There were no conflicts of interest.

### Participants

Sixty-five psychology students from the University of Hull took part in the study in return for a course credit. There were 32 males (Age: *M* = 23, SD = 4.8) and 33 females (Age: *M* = 25, SD = 8.7).

### Stimuli and equipment

Sixteen digitised photographs were selected from the Ekman and Friesen pictures of facial affect (Ekman & Friesen, 1976). The photographs were of 4 males (JJ, WF, GS, PE) and 4 females (MO, MF, NR, C) each displaying a happy and angry facial expression. The faces were presented at the centre of the screen at 5.5° of vertical angle and 3.6° of horizontal angle. Participants responded to the face pictures using a Logitech Attack 3 joystick. The joystick was fixed in position 65 cm from the computer monitor. The experiment was programmed in E-Prime.

### Design and procedure

Participants were instructed to classify the faces as either happy or angry as quickly and accurately as possible by either pushing or pulling the joystick. Participants completed a practice block of 16 trials followed by 2 main blocks with 128 trials in each block. The 16 practice trials consisted of 1 presentation of each of the 16 unique face images (8 happy, 8 angry). The main blocks consisted of 8 repetitions of the 16 unique face images leading to the creation of 128 trials. Response mapping (e.g., pull for angry, push for happy) was varied across the 2 main blocks. In one block, participants pushed the joystick for angry faces and pulled the joystick for happy faces. The response mapping was reversed in the second block—they were asked to push for happy and pull for angry. The order of the main blocks was randomised across participants using a random seed generated by the experimental software. Reaction times (RT) were recorded from onset of the face to initial movement of the joystick. Following the approach–avoidance task participants completed the Carver and White’s (1994) BAS and BIS Scales. The BIS/BAS consists of 24 statements designed to measure Behavioural inhibition (e.g., “I worry about making mistakes”) and 3 facets of Behavioural Activation namely, BAS Drive, BAS Fun Seeking and BAS Reward. Participants rated the statements on a 4-point scale (where 1 = “very true for me”, 2 = “somewhat true for me”, 3 = “somewhat false for me” and 4 = “very false for me”).

## Results

Two participants responded incorrectly on over 45% of trials and therefore, both were excluded from the data analyses. The full data set (including the excluded participants) and codebook can be found here https://osf.io/bmp2z/. Reaction times less than 200 ms and greater than 2500 ms were also removed prior to both RT analyses and diffusion modelling. This resulted in removal of 3% of the total number of trials. The data analysis was conducted in the R programming environment (R Development Core Team, 2018) using several packages (Kruschke & Meredith, 2017; Morey & Rouder, 2015; Wabersich & Vandekerckhove, 2014).

### Trait approach–avoidance characteristics (BIS and BAS scores)

Following previous research (Jorm et al. 1998), mean BIS scores for female participants were higher for female (*M* = 23.32, SD = 3.09) compared to male participants (*M* = 19.29, SD = 4.21), *F*(1,61) = 19.30, *p* < 0.001, $\eta _{p}^{2}$ = 0.24. Also, scores on the BAS scale were somewhat lower for female (*M* = 12.48; SD = 2.33) compared to male participants (*M* = 13.3; SD = 2.33), although the latter effect size was small, $\eta _{p}^{2}$ = 0.05, *F*(1,61) = 3.73, *p* = 0.05. The mean BIS scores were comparable (*M* = 22.0, SD = 3.4) to the 18–29 age group reported in a larger community-based sample by Jorm et al. (1998).

### Bayes factors

Analyses of RTs, error rates and EZ-diffusion model parameters were carried out using a 2 (expression) X 2 (response) X 2 (face-gender) X 2 (participant-gender) mixed ANOVA with participant-gender as the between subject factor. Evidence for a specific model term (e.g., the expression X response interaction) was quantified by calculating Bayes factors (BF) using the BayesFactor package (Morey & Rouder, 2015). Following recommendations (Jeffreys, 1961) Bayes factor values between 1 and 3.2 were interpreted as anecdotal evidence against the null hypothesis; values between 3.2 and 10 as moderate evidence; values between 10 and 100 as strong evidence; and values > 100 as decisive evidence. Each model term was compared to a null model that included participant as a random variable. The reason for using Bayes factors was to guide statistical inference toward the more substantive effects. Finally, for all follow-up tests of differences I used a one-sample Bayesian *t* tests to estimate both a standardised effect sizes d*z* (*µ* − 0/*σ*) and the 95% highest density interval (HDI) around the effect size difference from zero. I used the BEST package (Kruschke & Meredith, 2017) for the latter calculations.

### Reaction times

For median correct RTs, 3 ANOVA model terms received strong support relative to the null model: (1) the main effect of expression (BF = 2,557,763), (2) the expression X response interaction (BF = 24,819,159) and (3) the three-way expression X response X participant gender interaction (BF = 328). Model terms that received moderate support relative to the null are the participant-gender X expression X response interaction (BF = 8.72) and the main effect of response (BF = 6.82).

**Fig. 1:**
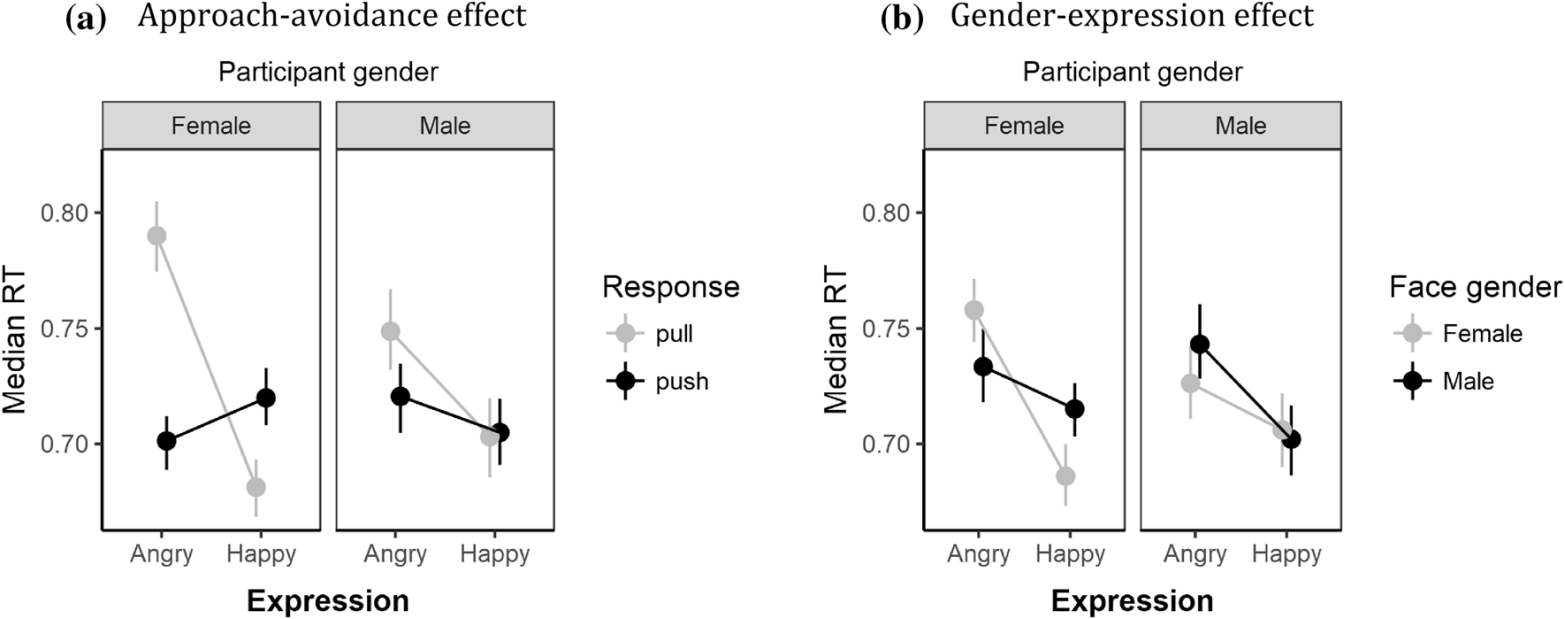
Means of the median correct RTs for female and male participants as a function of expression and response (**a**—top) and expression and face-gender (**b**—bottom). Error bars are bootstrapped standard errors

As shown in Fig. 1, results indicate that both the gender-expression bias (Fig. 1b) and approach–avoidance bias (Fig. 1a) are larger in magnitude for female compared to male participants. Follow-up tests support this observation. Specifically, focussing on the participant-gender X expression X response interaction, simple interaction effect analyses showed that the expression X response pattern received strong support relative to the null model for female (BF = 82,701,289,305), but not male participants (*BF* = 0.57). Following Rotteveel and Phaf (2004), female participants showed the approach–avoidance effect—they were faster to push (vs pull) the joystick in response to angry faces (*d*_*z*_ = − 0.88; 95% HDI [− 1.33, − 0.46]) and conversely, faster to pull (vs pull) the joystick in response to happy faces (*d*_*z*_ = − 0.60; 95% HDI [− 1.01, − 0.21]). Male participants were faster to respond to happy compared to angry expressions (*d*_*z*_ = − 0.63; 95% HDI [− 1.10, − 0.18]). In short, for the approach–avoidance bias, the results appear to replicate moderation of the effect by participant gender reported by Solarz (1960).

Focussing on the participant-gender X expression X face-gender interaction, the expression X face-gender interaction received strong support for female (BF = 11) but not male participants (BF = 0.32). Follow-up analyses showed that for female participants, median RTs were faster for male angry faces compared to female angry faces (*d*_*z*_ = − 0.69; 95% HDI [− 1.12, − 0.27]) and also, faster for female happy faces compared to male angry faces (*d*_*z*_ = − 0.96; 95% HDI [− 1.45, − 0.47]).

### Analyses of proportion correct

A significant Shapiro–Wilk’s test indicated that the mean proportion of correct responses was not normally distributed (*p* < 0.001). Arc sine transformation of the proportion correction failed to correct for non-normality (Shapiro–Wilk’s test; *p* < .001). Distributional analyses indicated an adequate fit when the correct responses were modelled as a binomially distributed random variable and therefore, a regression model with a binomial response function was chosen to model the data. Specifically, to account for repeated measurements within subjects, a multilevel logistic regression model was fit to the number of correct responses using generalised estimating equations with an exchangeable covariance matrix.

The predictors were sum-coded to make the analyses comparable to the more frequently used ANOVA. Also, like ANOVA, all predictors were included in a single model. There were main effects of expression (Wald statistic = − 0.0109, *p* = 0.0006) and response (Wald statistic = 4.37, *p* = 0.03) and a face-gender X expression interaction (Wald statistic = 13.3, *p* = 0.0002). Analyses of the interaction effect showed that the effect of face-gender was significant for angry faces (*z* = 2.74, *p* = 0.006) but not happy faces (*z* = − 1.46, *p* = 0.14). In summary, analyses of accuracy show that (1) accuracy rates were (overall) higher for happy expressions (*M* = 95%) compared to angry expressions (*M* = 93%) and (2) for angry faces specifically, accuracy was higher for male faces (*M* = 94%) compared to female faces (*M* = 92%). The effect of response indicated a very small increase in accuracy for push responses (*M* = 94.8%) compared to pull response (*M* = 94.0%).

### Exploratory analyses of BIS and BAS scores and reaction times

Further analyses were conducted to establish whether the gender-expression and approach–avoidance reaction time biases are associated with individual differences in BIS and BAS scores. Such analyses are challenging within the context of ANOVA or ANCOVA and therefore, I used multilevel regression. Distributional analyses indicated an adequate fit when the reaction times were modelled as an ex-Gaussian distributed random variable and therefore, a regression model with an ex-Gaussian response function was chosen to model the data. Specifically, I estimated 4 separate regression models for each of BIS/BAS subscales (BIS, BAS reward, BAS Drive, and BAS fun-seeking). Treatment (dummy) coding was used for all categorical variables (expression, response, face-gender). BIS, BAS reward, BAS fun-seeking and BAS drive scores were mean centred and scaled to 2 standard deviations (Gelman, 2008) to facilitate interpretation of interactions. All models included random intercepts (by-participants) and random slopes for expression, response and face-gender. I did not estimate a correlation between the random effects. Each model included 2 separate, 4-way interaction terms. For example, the model designed to examine the influence of BIS scores included an expression X face-gender X participant gender X BIS interaction term and an expression X face-gender X participant gender X BIS interaction term.

There was a single, significant 4-way interaction. Specifically, for model that included the mean-centred BIS scores, there was a significant positive significant slope, *β*_(happy X pull X female X BIS)_ = 0.12, *t* = 6.23, *p* < 0.00001. The positive slope (0.12) of this interaction indicates that the tendency for female participants (at mean levels of BIS) to pull the joystick faster in response to happy faces (*β*_(happy X pull X female X BIS)_ = − 0.09, *t* = − 9.53, *p* < 0.00001) decreased in female participants who reported high levels of behavioural inhibition. In other words, for female participants, the approach–avoidance effect decreased with increases in behavioural inhibition. All other effects failed to reach statistical significance (all *p*s > 0.1).

### EZ-diffusion model analyses

The estimated drift rates, boundary separation values and non-decisions times were analysed in 3 mixed Bayesian ANOVAs; 2 (expression) X 2 (response) X 2 (face-gender) X 2 (participant gender) with participant gender as the between subject variable.

### Drift rates

**Fig. 2:**
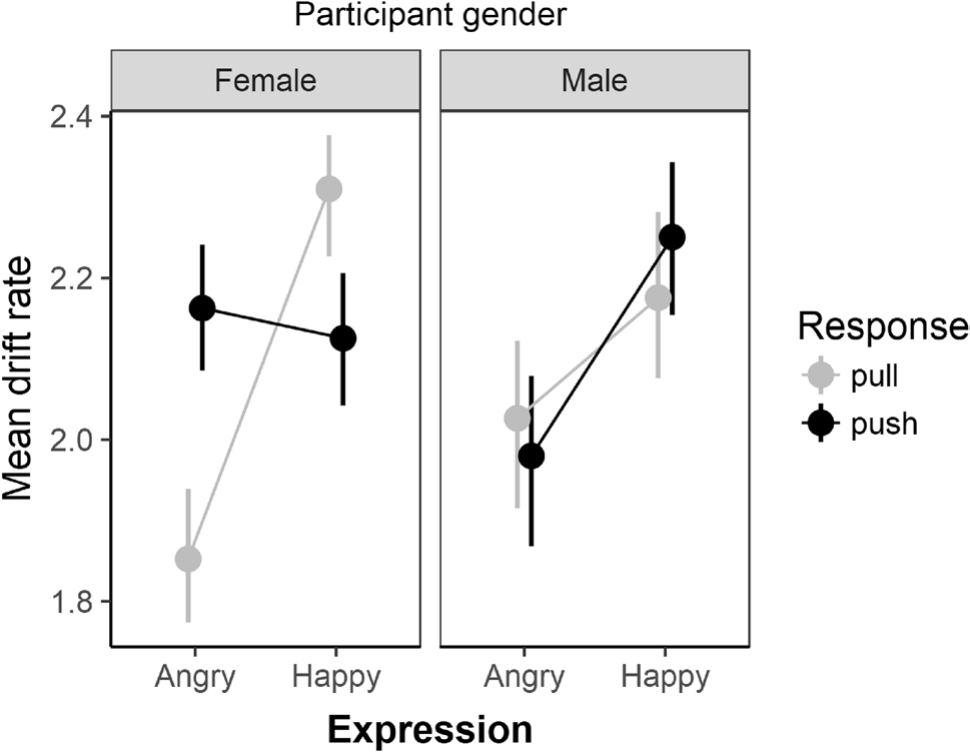
Mean drift rates as a function of expression and response for female and male participants separately. Error bars are bootstrapped standard errors

In Fig. 2, I have plotted the mean drift rates as a function of expression and response for female and male participants separately. ANOVA supports the observed pattern namely higher drift rates when female participants were required to push (vs pull) in response to angry faces and (to a lesser extent) the reverse pattern for happy faces. Specifically, for drift rates, 2 ANOVA model terms received strong support relative to the null model: (1) the main effect of expression (BF = 738) and (2) the three-way expression X response X participant gender interaction (BF = 20). To analyse the three-way interaction, I calculated expression X response simple interaction effects for males and females separately. Simple interaction effects analyses supported the inclusion of the expression X response term for females, BF = 103 but not for males (BF = 0.26). Further analyses support the pattern of differences indicated in Fig. 2 namely, higher drift rates when female participants were required to push (*M* = 2.16; SD = 0.66) vs pull (*M* = 1.85, SD = 0.66) in response to angry faces and the reverse pattern for happy faces—higher drift rates when female participants were required to pull (*M* = 2.31; SD = 0.61) vs push (*M* = 2.12; SD = 0.65). Specifically, for female participants, Bayes factor analyses indicated moderate evidence supporting the push minus pull difference from zero for angry faces (BF = 5.67; *d*_*z*_ = 0.56; 95% HDI [0.17, 0.94]) and weaker support (BF = 1.13) for the pull minus push difference compared to zero for happy faces (*d*_*z*_ = 0.36; 95% HDI [0.001, 0.78]). For male participants, mean drift rates were higher for happy compared to angry expressions (BF = 1.49; *d*_z_ = 0.39, 95% HDI [0.0, 1.31]).

### Non-decision times

**Fig. 3:**
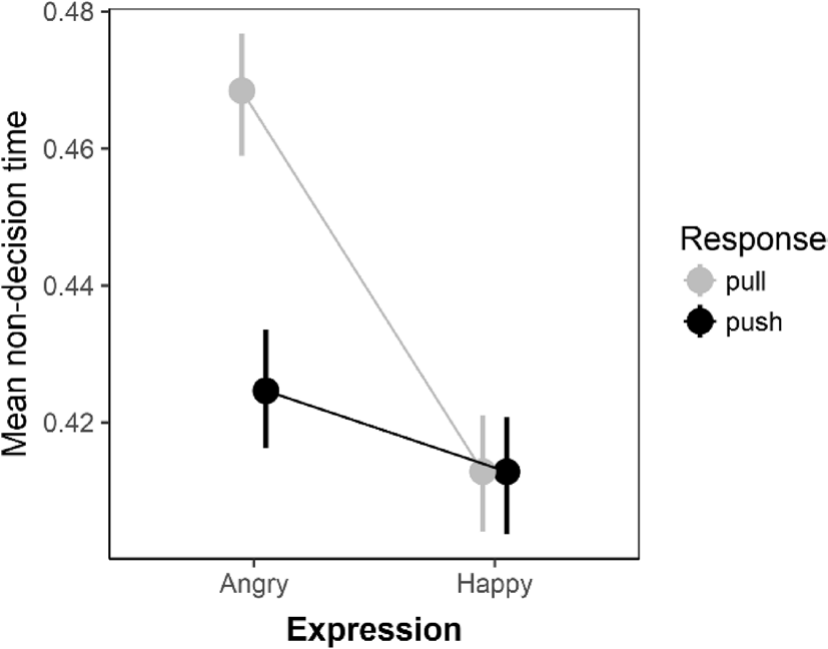
Mean non-decision times as a function of both expression and response and expression. Error bars are bootstrapped standard errors

For non-decision times, Bayesian ANOVA indicated four key effects with moderate to strong evidence relative to the null model: (1) a main effect of expression (BF = 732), (2) an expression X face-gender interaction (BF = 16), (3) an expression X response interaction (BF = 5) and (4) an expression X face-gender X participant gender interaction (BF = 275). The expression X response interaction is displayed in Fig. 3. Follow-up tests support the observed pattern of means; mean non-decision times were lower for push vs pull responses to angry faces (BF = 34; *d*_z_ = − 0.44, 95% HDI [− 0.17, − 0.71]), whereas for happy faces, non-decision times were similar for push and pull responses (BF = 0.13; *d*_z_ = 0.07, 95% HDI [− 0.19, 0.33]).

**Fig. 4:**
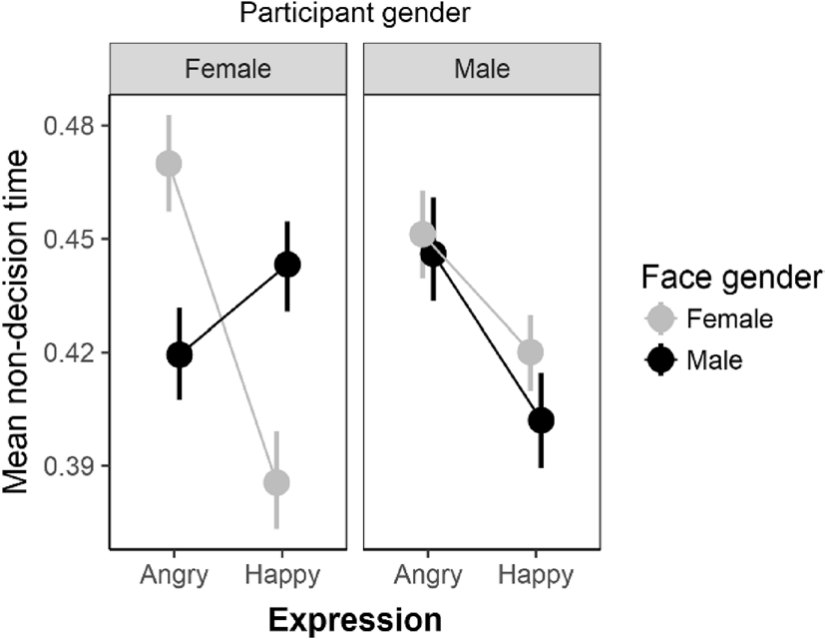
Mean non-decision times as a function of expression and face-gender for female and male participants separately. Error bars are bootstrapped standard errors

The expression X face-gender X interaction is displayed in Fig. 4. For female participants, there was substantial evidence favouring the expression X face-gender relative to the null model (BF = 12,680). Follow-up tests support the pattern in Fig. 4—faster non-decision times for angry-male faces compared to angry female faces (BF = 42; *d*_z_ = 0.71, 95% HDI [0.28, 1.15]) and also, faster non-decision times for happy female compared to happy male faces (BF = 360; *d*_z_ = 0.80, 95% HDI [0.38, 1.21]).

### Boundary separation

**Fig. 5:**
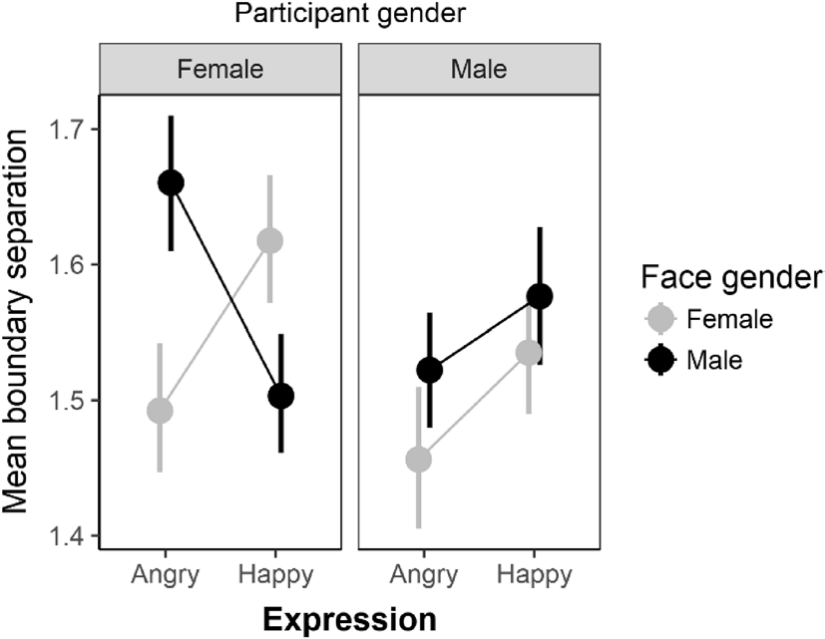
Mean boundary separation values as a function of expression and face-gender for female and male participants separately. Error bars are bootstrapped standard errors

For boundary separation values, Bayesian ANOVA indicated strong evidence for the expression X face-gender interaction (BF = 17) and moderate evidence for an expression X face-gender X participant gender interaction (BF = 3.66). As shown in Fig. 5, for females there was a very strong (BF = 181) expression X face-gender interaction with higher boundary settings for male angry faces compared to female angry faces (BF = 6.96, *d*_z_ = 0.52, 95% HDI [0.134, 0.94]). For happy faces, the female minus male difference was smaller in magnitude (*d*_z_ = 0.44, 95% HDI [0.07, 0.84]) and evidence for the difference vs the null model was moderate (BF = 2.89). For male participants all evidence against the null was weak for all effects (all BFs < 0.1). In summary, analysis of boundary separation values indicates that female but not male participants, were relatively more cautious when responding to male angry, and to a lesser extent, female happy faces.

### Assessment of model fit

**Fig. 6:**
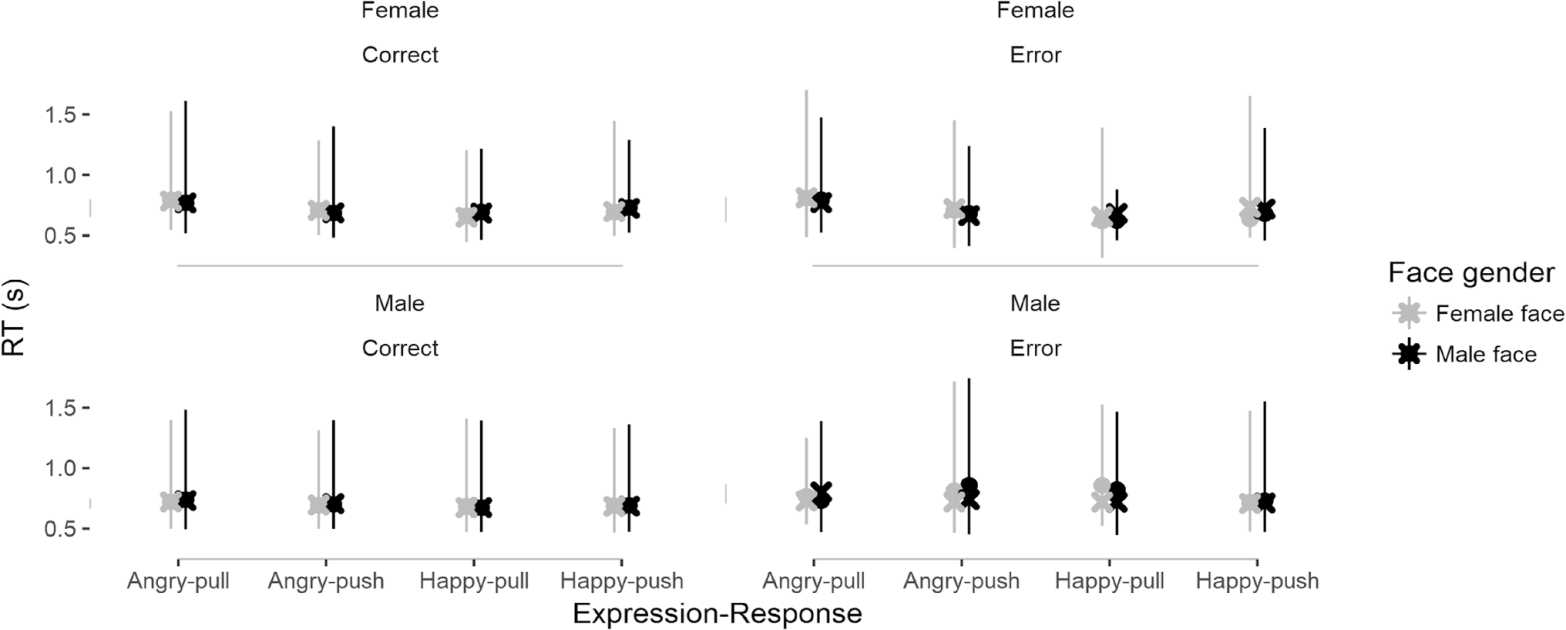
Median of the predicted (crosses) and observed RTs (circles) for every possible combination of expression, response, face-gender and participant gender. Error bars extend from the 5th to 95th percentile of the observed data

I assessed model fit using data simulation (Gelman and Hill, 2007). Specifically, I used the RWiener package (Wabersich & Vandekerckhove, 2014) to generate 10,000 RT trials from the fitted parameter estimates of drift rates, boundary separation values and non-decision times for each participant for each combination of face-gender, response and expression. In Fig. 6, I have plotted the median of the predicted RTs (crosses) for each expression, response, face-gender, participant gender against the median of the observed data (circles). Error bars extend from the 5th to 95th percentile of the observed data. The predicted RTs appear to fit the data well although the fit is noticeably worse for error responses (and particularly for male participants) where there are a low number of observed responses.

## Discussion

RT analyses and diffusion model results contribute to our understanding of face processing in two key ways. The first contribution is to show that participant gender differences moderate both the approach–avoidance and gender-expression biases. Both effects were larger in magnitude for female participants. The second contribution of this study is to show—using diffusion model analyses—that the two biases are mediated by separate processes.

For the RT analyses, both the gender-expression recognition and approach–avoidance biases were larger in magnitude for female participants. Specifically, for female participants, there was: (1) an approach avoidance effect—RTs were faster when avoiding angry faces (by pushing a joystick) and approach-happy faces (by pulling the joystick) and (2) a gender-expression effect—RTs were faster for angry-male and happy-female faces relative to their opposite gender pairs. The enhanced RT effect for female participants is consistent with the pioneering work of Solarz (1960) and, with a later study by Rotteveel and Phaf (2004) that tested only female participants.

Despite Solarz’s initial findings and the current study, other studies have not reported participant gender differences (Krieglmeyer & Deutsch, 2013; Marsh et al., 2005; Seidel et al., 2010; Veenstra et al., 2017). Why was the current study sensitive to participant gender differences? The current research may have been especially sensitive to participant-gender differences in the approach–avoidance bias because the basic design fits the conditions highlighted most likely to lead to a recording of approach–avoidance bias. In brief, a recent study (Paulus & Wentura, 2016) showed that the direction of the approach–avoidance effect for facial expressions depends on the choice of comparison expression. When sad expressions were paired with happy expressions they elicited avoidance reactions. When sad expressions were paired with angry expressions they elicited approach reactions. The authors suggest that under the latter conditions reactions were based on social meaning: Faces elicited approach because sadness is considered a request for help (Horstmann, 2003). In the current experiment I used evaluatively opposite pairs and therefore, considering the findings of Paulus & Wentura (2016), the conditions were optimal for recording an approach-happy, avoid-angry effect. A further point is that even though participant gender differences have not been consistently recorded across approach–avoidance tasks in previous research there is evidence for participant gender differences in identification RTs for facial expressions (Thompson & Voyer, 2014). For example, one study (Hampson, van Anders, & Mullin, 2006) found that women were faster than men at recognising both positive and negative facial expression, although the sex difference was larger for negative expressions. Overall, the current findings and the wider literature support the continued inclusion of participant gender as a variable in the analysis of the approach–avoidance and gender-expression biases.

In agreement with the results of larger community-based samples (Jorm et al., 1998) in the current study, trait avoidance scores measured using the BIS subscale were significantly higher in female compared to male participants. Considering this relationship, a larger sample would be needed to establish whether self-reported behavioural inhibition scores independently (of participant gender) contribute to the approach–avoidance and face-gender biases. Nonetheless, for the current I attempted to establish the extent to which BIS and BAS scores are associated with the face-gender and approach–avoidance biases within the male and female groups. Regression model results showed that for female participants, higher BIS scores were associated with a reduction in the approach–avoidance effect. Separate research findings show that high behavioural inhibition (measured using the BIS subscale) is associated with high current depression ratings and therefore, the current decrease in the approach–avoidance effect for female participants reporting high levels of behavioural inhibition may reflect a general decrease in emotional reactivity.

Previous studies have not reported an association between BIS scores and the approach–avoidance effect (van Peer et al. 2010; Struijs et al., 2017). One reason why this effect might have been reported here and not in previous research is that in the current research I selected the ex-Gaussian distribution to model reaction times. Specifically, reaction times were modelled as originating from the mean of the (first) Gaussian moment of the ex-Gaussian distribution. In contrast to the EZ-Diffusion Model, parameters of the ex-Gaussian are not tied to underlying psychological process—the model provides a good descriptive, rather than a cognitive process account of reaction times (Heathcote, Popiel, & Mewhort, 1991). Therefore, the current research opens a further avenue of research—using distributional analyses to model individual differences in approach–avoidance tendencies.

### Diffusion model results

The diffusion model results suggest that the gender-expression and approach–avoidance biases are mediated by separate processes. Specifically, for female participants, the gender-expression bias was mediated by changes in both non-decision times and response caution whereas the approach–avoidance bias was mediated by changes in evidence accumulation. The effect of approach–avoidance-conditions on drift rates extends a recent study (Krypotos et al., 2015) where the authors reported higher drift rates (and faster median RTs) when participants were required to avoid a punishing stimulus and approach a rewarding stimulus. Here, I show that the effect on drift rates is also found for facial expressions. Specifically, drift rates were higher when participants were required to push (vs pull) to angry faces and higher when they were required to pull (vs push) in response to happy faces. Again, following the general pattern for the results reported here, the effect was larger for female participants.

Beyond drift rates, the current results also extend previous research by showing that non-decision times are also affected in avoidance-compatible conditions. Specifically, for all participants—irrespective of participant gender—non-decision times were reduced for angry faces in the push compared to the pull condition. The pattern was reversed for happy faces although Bayes factor analyses indicated that the evidence in favour of a difference was weak. This effect for non-decision times indicates that either response execution or encoding times were reduced when participants avoided angry faces. Given the status of the approach–avoidance effect as an action bias, one explanation is that the effect reflects faster response execution times.

For the gender-expression recognition bias, the analyses of RTs and accuracy (for female participants) matched those reported by Becker et al.—specifically, reaction times were faster and accuracy rates were higher for angry-male and happy-female faces compared to the opposite sex pairs. The EZ-diffusion model offers insight into the possible process responsible for this effect. Specifically, the results showed that the expression X face-gender pattern was found for both non-decision times and boundary separation values. Recent research indicates that the gender-expression recognition bias operates at a perceptual level—perhaps engaging an evolved gender-recognition system. Therefore, although a response execution explanation cannot be ruled out by the current results, the reduction in non-decision times for angry-male and happy-female faces in female participants is at least suggestive of a gender-based encoding bias.

The diffusion model results provide further insight into the effect of face-gender on facial expression recognition accuracy. Specifically, participants were relatively more cautious—boundary separation values were higher—when responding to angry-male faces and to a lesser extent happy-female faces. This is another way in which the diffusion model results extend beyond the analysis of accuracy and RT as separate variables because such analyses do not directly estimate the relationship between accuracy and RTs.

To interpret a change in decision-making caution due to changes in the stimulus properties, it is necessary to make a specific assumption concerning the sequence of processing with the approach–avoidance task. Specifically, a key assumption of the diffusion model is that thresholds (indexed by boundary separation values) and starting points are fixed before the diffusion process starts. Therefore, to record an effect of face-gender on either decision thresholds or the starting point (initial bias) it is necessary to make an additional assumption. Specifically, gender must be the first information processed by the participant (for a similar argument applied to a different task see; Pleskac, Cesario, & Johnson, 2017). This is indeed consistent with the decrease in non-decision times for angry-male and happy-female faces. Taken together one interpretation of the reduction in non-decision times and increase in boundary separation rate is that face-gender led to enhanced perceptual encoding followed by the setting of higher thresholds for angry-male and happy-female faces. In other words, a perceptual bias followed by a strategic adjustment of thresholds. So this would translate into an early identification of face-gender (“its male”) followed by (before diffusion process starts) a change in caution (“its likely to be angry and therefore I will require a lot of evidence to be convinced otherwise”). This is relevant to wider theory in this area because it is consistent with the operation of an early perceptual bias in conjunction with strategic setting of thresholds based on stereotypes. In short, these findings show how future work can target manipulations at processes that give rise to these influences.

In summary, the current study supports the idea that both approach–avoidance and gender-expression recognition bias are larger in magnitude for female participants. Further, diffusion model results suggest possible mechanisms responsible for such effects and provide a clear path for future research—studying how we process facial expressions by jointly modelling accuracy and RTs using the diffusion model.

## Electronic supplementary material

Below is the link to the electronic supplementary material.

Supplementary material 1 (CSV 704 KB)
